# Expression of concern: ‘Investigating the bioavailability of graphene quantum dots in lung tissues via Fourier transform infrared spectroscopy’ by Tabish *et al*.

**DOI:** 10.1098/rsfs.2025.0051

**Published:** 2025-08-20

**Authors:** Interface Focus Editorial Office

**Affiliations:** ^1^ The Royal Society, London, UK

**Keywords:** graphene quantum dots, bioavailability, lung tissues

*Interface Focus*. **8**, 20170054 (Published online 20 April 2018). (https://royalsocietypublishing.org/doi/10.1098/rsfs.2017.0054).

Following the publication of ‘Investigating the bioavailability of graphene quantum dots in lung tissues via Fourier transform infrared spectroscopy’ (published: 20 April 2018), the Editorial team have concerns regarding the integrity of some of the figures shown in the study.

We are issuing an expression of concern here and investigating this as a matter of urgency; we will notify readers as to the results of our investigation as soon as possible.

